# The Route of Misoprostol in Reducing Blood Loss for Transperitoneal Myomectomies: A Systematic Review and Meta-analysis

**DOI:** 10.1097/og9.0000000000000151

**Published:** 2026-02-19

**Authors:** Nadi Kaonga, Emma Patterson, Mariana C. Sanchez-Medina, Tionne Pete, Natalie Rilo, Erin Reardon, Scott Eugley, Cara Frankenfeld, Toby Fitzgerald

**Affiliations:** Department of Gynecology and Obstetrics, Emory University School of Medicine, Atlanta, Georgia; Tufts University School of Medicine, MaineHealth Medical Center, Portland, Maine; Department of Obstetrics and Gynecology, Kaiser Oakland, Oakland, California; Department of Pediatrics, Medical College of Georgia, Augusta University, Augusta, Georgia; Department of Obstetrics and Gynecology, University of Colorado, Denver, Colorado; Woodruff Health Sciences Center Library, Emory University, Atlanta, Georgia; and Center for Interdisciplinary Population & Health Research and Department of Obstetrics and Gynecology, MaineHealth, Portland, Maine.

## Abstract

**OBJECTIVE:**

To compare the efficacy of different routes of misoprostol administration (sublingual, vaginal, rectal) in reducing intraoperative blood loss, which remains a concern for the surgical management of uterine leiomyomas.

**DATA SOURCES:**

The protocol was registered in PROSPERO (CRD42025637576). Following PRISMA (Preferred Reporting Items for Systematic Reviews and Meta-analysis) guidelines, we searched several databases (Ovid Medline, Embase, Scopus, Web of Science, Cochrane, Global Health, Global Index Medicus, ClinicalTrials.gov) through June 2024.

**METHODS OF STUDY SELECTION:**

The references were screened and data were abstracted in Covidence for studies meeting inclusion criteria. Title abstract screening was conducted, followed by full-text review and then data abstraction. Analyses were conducted with R.

**TABULATION, INTEGRATION, AND RESULTS:**

There were 328 studies identified in the initial search. Of those, 26 studies were included for analysis, but only 20 were able to undergo network meta-analysis against the primary outcome of interest (blood loss). Rectal misoprostol administration was associated with the largest mean reduction in intraoperative blood loss (mean difference −152.43 mL, 95% CI, −228.43 to −76.44, *P*<.0001), followed by vaginal administration (mean difference −69.46 mL, 95% CI, −122.11 to −16.82, *P*=.010). Sublingual administration was not statistically significant (mean difference −92.13 mL, 95% CI, −234.95 to 50.70, *P*=.206). Across other key outcomes (eg, blood transfusion, operative time), rectal misoprostol generally outperformed vaginal and sublingual modes of administration in our network meta-analysis.

**CONCLUSION:**

Route of misoprostol administration does matter in reducing blood loss during abdominal myomectomy with rectal administration favored, although the data are limited for sublingual administration.

**SYSTEMATIC REVIEW REGISTRATION:**

PROSPERO, CRD42025637576.

## Abstract 2

Rectal misoprostol is associated with reduced blood loss in transperitoneal myomectomies compared to other modes of administration.

Leiomyomas are solid tumors composed of smooth muscle and connective cell–type growths that can present in several locations.^1^ Uterine leiomyomas are among the most common benign gynecologic tumors, affecting up to 70–80% of women by the age of 50.^2^ The International Federation of Gynecology and Obstetrics has developed a standardized subclassification system to describe uterine leiomyoma based on location in relation to the endometrial and serosal surfaces.

Symptoms of uterine leiomyomas may vary, depending on their International Federation of Gynecology and Obstetrics classification and size.^2^ The most common symptoms of uterine leiomyomas include abnormal uterine bleeding caused by leiomyoma and bulk-related symptoms such as pelvic pain, dyspareunia, bloating, urinary frequency, and constipation. Menstrual bleeding can be prolonged and heavy and is associated with anemia. Several treatment options exist for management of uterine leiomyomas, including expectant, medical, interventional, and surgical therapies.^1^ When surgical treatment options are being discussed, patient-specific symptoms and severity, as well as desire for uterine conservation and possible future pregnancy, should be considered. Goals of treatment should be individualized according to symptomatology, severity, and wish for uterine preservation and future fertility. Significant intraoperative blood loss is a major concern during myomectomy, often necessitating blood transfusion and increasing the risk of perioperative morbidity.^1^ To mitigate blood loss, various agents, given both preoperatively and intraoperatively, have been explored.

Misoprostol, a synthetic prostaglandin E1 analog, is well studied as a useful and effective option because of its uterotonic properties and affordability.^3,4^ Emerging evidence supports the use of misoprostol at the time of myomectomy in decreasing blood loss. Misoprostol is thought to increase myometrial contraction and to have a vasoconstrictive effect on the uterine artery, thus decreasing blood loss.^2^

Although multiple studies have demonstrated that misoprostol can reduce intraoperative blood loss in abdominal myomectomy, a critical gap remains in our understanding of whether the route of administration affects its efficacy.^5^ Misoprostol can be administered through several routes—sublingual, vaginal, and rectal—each with differing pharmacokinetics.^6^ Sublingual administration offers rapid absorption and peak plasma levels; vaginal administration provides prolonged bioavailability; and rectal administration is often preferred for its ease of use and reduced gastrointestinal side effects.^6^ However, it remains unclear which route provides the most effective hemostatic benefit in the context of an abdominal myomectomy.

This systematic review and meta-analysis aims to synthesize the existing evidence on the use of misoprostol for reducing intraoperative blood loss during transperitoneal myomectomy, with a specific focus on comparing the efficacy of different routes of administration. This will build on previous systematic reviews and meta-analyses on this topic more broadly,^7–15^ as well as a prior systematic review that had explored this issue; however, only five studies were included at that time,^11^ and they were not able to evaluate differences across routes of administration. By addressing this gap, our study could inform clinical practice and optimize surgical outcomes for patients undergoing myomectomy.

## SOURCES

After we developed the search strategy and terms, we systematically searched the following databases: Ovid Medline, Embase, Scopus, Web of Science, Cochrane, Global Health, Global Index Medicus, and ClinicalTrials.gov. The full search strategy is available in Appendix 1, available online at http://links.lww.com/AOG/E535.

## STUDY SELECTION

The study protocol was registered in PROSPERO,^16^ and PRISMA (Preferred Reporting Items for Systematic Reviews and Meta-analyses) was used to guide the overall approach. The review was carried out from June 2024 to March 2025.

Studies were eligible for inclusion on adult populations with female reproductive organs undergoing myomectomy who received misoprostol placed vaginally compared with misoprostol administered sublingually or rectally. Hysteroscopic myomectomies were excluded because misoprostol is unlikely to be placed vaginally in this procedure. Experimental study designs were included. There were no requirements for a minimum number of study participants.

Search results were deduplicated in Endnote 21 before being uploaded to Covidence for screening and data extraction.^17^ References were examined separately to identify any additional studies for review. The studies were then uploaded into Covidence. Titles and abstracts were screened according to the inclusion and exclusion criteria of the protocol, with two reviewers required to screen each title and abstract. Conflicts were resolved through discussion. After this, full-text reviews were conducted, again with two reviewers screening each articles and any discrepancies adjudicated.

On completion of the full-text reviews, data were abstracted from each of the articles with the use of a standardized data collection form in Covidence. The form included the following: study identifiers, methods, inclusion and exclusion criteria, baseline characteristics of study participants, details on intervention(s) and placebo/control, and outcomes data. Two reviewers were required to abstract data independently per article; quality reviews of the data were conducted after completion of two entries. After ensuring that all reviewers had accurate data abstraction, we conducted the remainder of the extraction. After all entries were completed, we conducted consensus reviews of the data, and any further discrepancies were resolved through discussion. An attempt was also made to reach out to authors on record for 10 studies with missing data, but no responses were received.

The risk of bias for each study was then assessed with a standardized form in Covidence, which draws from the Cochrane Risk of Bias and includes sequence generation, allocation concealment, blinding of participants and personnel, blinding of outcome assessors, incomplete outcome data, selective outcome reporting, and other sources of bias. Any discrepancies were adjudicated.

Data were downloaded from Covidence as a Comma-Separated Values and then imported into R 4.2.1 for cleaning and analysis; the pooled κ agreement was calculated. The dataset was additionally screened for completeness and harmonized for network meta-analysis. For completeness, the continuous outcome variables (intraoperative blood loss, operative time, hemoglobin change, and hematocrit change) required number (sample size) and mean±SD, and the binary transfusion events variable required number (sample size) and number of events. For harmonization, consistency of outcome units (grams per deciliter for hemoglobin, milliliter for blood loss, minutes for operating time, etc) was reviewed and direction of effect was standardized (eg, greater blood loss equals worse outcome, decrease in hemoglobin from baseline to after the procedure equals worse outcome). For change outcomes (hemoglobin and hematocrit) with only baseline and postoperative SDs, the SD change was approximated with the following formula: SD_change=√(SD_baseline^2^+SD_postop^2^−2·*r*·SD_baseline·SD_postop). Because no change SDs were reported, we set *r*=0 (conservative).

Misoprostol interventions were mapped to routes of administration (sublingual, vaginal, rectal) with no misoprostol as the reference. A frequentist network meta-analysis using the graph-theoretical approach in the netmeta package was performed, fitting both common-effects and random-effects models.^18^ Between-study variance (τ^2^) was estimated and heterogeneity and inconsistency were calculated with the Cochran Q and *I*^2^, partitioning Q into within-design (heterogeneity) and between-design (inconsistency) components. Treatment rankings were assessed with *P* scores. Multiarm trials were handled with the netmeta built-in framework to account for correlations among the comparisons. Continuous outcome variables were analyzed as mean differences with corresponding 95% CIs. Risk ratios with 95% CIs were calculated for dichotomous outcomes (eg, transfusion events). Common-effects and random-effects results are reported.

## RESULTS

**Fig. 1.**
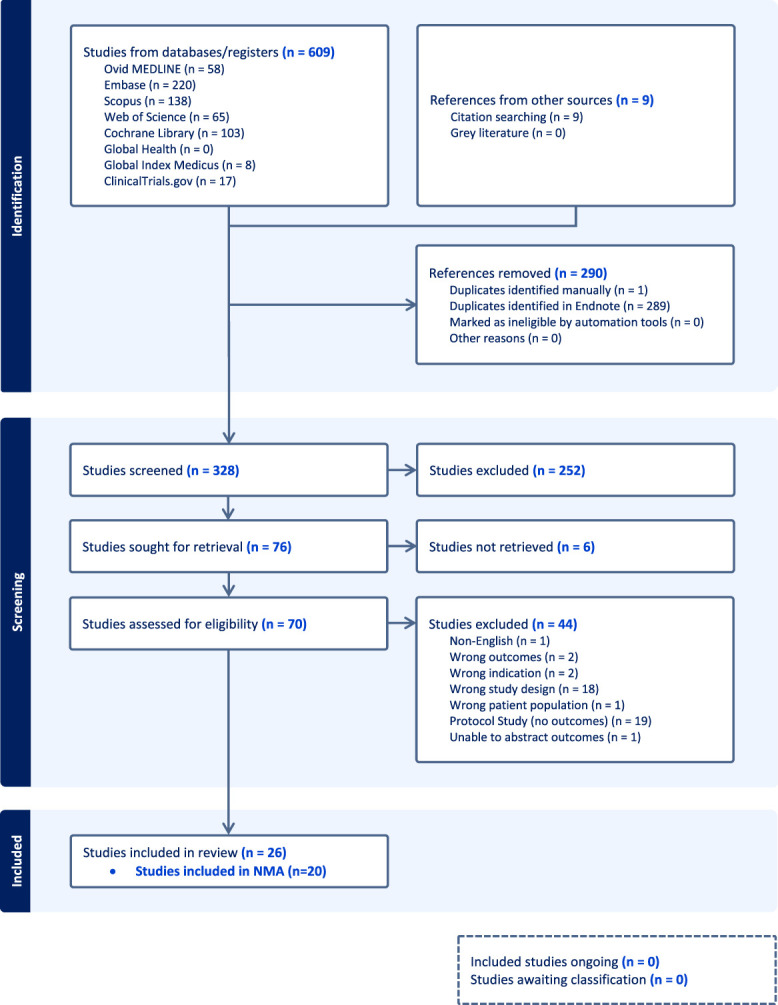
PRISMA (Preferred Reporting Items for Systematic Reviews and Meta-Analyses) flow diagram. NMA, network meta-analysis.

There were 609 studies identified through the database search, and an additional 9 references were identified after a search through citations. After the removal of duplicates, 328 studies were eligible for title and abstract screening. Twenty-six studies were ultimately included for full review and data abstraction (Fig. 1).^19–44^ Six studies were excluded because of missing SDs, leaving 20 studies included in the network analysis against the primary outcome of interest.^21–27,29–34,36–41,44^ The pooled κ agreement was 0.53.

*Kaonga. Route of Misoprostol: Meta-analysis. O&G Open 2026*.

Eleven countries were represented across the studies. The majority of studies (n=10, 38%) took place in Egypt.^20,21,23–25,27,30,35,39^ All but two studies occurred in a single site; the two multicenter studies were in Nigeria. Studies took place over 2 months to just over 4 years with the median duration of 15.25 months (interquartile range 11.25 months) (Table 1).

**Table 1.** Summary Table of Studies

First Author, Year	Study Type	Randomization	Allocation Concealment	Blinding	Country	Site	Dates	Route of Myomectomy	Sample Size (n)	Interventions		Blood Loss mean±SD (mL)		Operative Time, mean±SD (min)	
										Intervention A	Intervention B	A	B	A	B
Abbas, 2019	RCT	1:1 random allocation		Double	Egypt	Single			80	200 micrograms and placebo sublingual misoprostol at 3 and 1 h preoperatively (n=40)	400 micrograms sublingual misoprostol at 3 and 1 h preoperatively (n=40)	560.0±105.2	373.3±55.6	111.2±6.3	91.3±5.7
Abbas, 2020	RCT			Double	Egypt	Single, university	March 2017–December 2019	Abdominal	60	400 micrograms rectal misoprostol 1 h preoperatively (n=30)	400 micrograms sublingual misoprostol 1 h preoperatively (n=30)	247.44±106.04	256.17±116.27	N/A	N/A
Abdel-Hafeez 2015	RCT	Random number generator, 1:1 allocation	Sealed envelopes	Double	Egypt	Single, university	March 2013–May 2014	Abdominal	63	Rectal placebo 1 h preoperatively (n=25)	400 micrograms rectal misoprostol 1 h preoperatively (n=25)	874.0±71.5	574±194.8	94.8±22.8	76.8±15.8
Afolabi, 2019	RCT	Random number generator	Sealed envelopes	None	Nigeria	Single, university	June 2016– May 2017	Abdominal	120	Tourniquet intraoperatively (n=40)	400 micrograms vaginal misoprostol 1 h preoperatively (n=40)	848.40±588.85	931.89±602.13	102.13±38.76	109.48±34.90
Alhalaby, 2021	RCT				Egypt	Single, university	October 2019– October 2020	Abdominal	50	Vaginal placebo 1 h preoperatively (n=25)	400 micrograms vaginal misoprostol 1 h preoperatively (n=25)	404.4±87.18	308±32.66	78.6±10.6	56.8±3.12
Alhameedi, 2021	RCT	Open randomized sampling method		None	Egypt	Single, university	August 2020–February 2021	Abdominal	60	Tourniquet intraoperatively (n=30)	400 micrograms vaginal misoprostol 1 h preoperatively (n=30)	375±96.3	400±78	47.5±4.7	53±6.9
Ali, 2019	RCT			None	Egypt	Single, university		Abdominal	36	Tourniquet intraoperatively (n=18)	400 micrograms vaginal misoprostol 1 h preoperatively (n=18)	408.3±94.3	493.9±125.2	88.9±10.8	98.1±13.3
Celik, 2003	RCT	Randomized numbers table		Double	Turkey	Single, university	January 10, 2000–January 10, 2002	Abdominal	25	Vaginal placebo 1 h preoperatively (n=13)	400 micrograms vaginal misoprostol 1 h preoperatively (n=12)	621±121	472±77	58±8.8	48.5±7.4
El Sharkwy, 2016	RCT	Random number generator	Sealed envelopes	None	Egypt	Single, university	March 2013–April 2015	Abdominal	104	Tourniquet intraoperatively (n=52)	400 micrograms rectal misoprostol 1 h preoperatively, perivascular vasopressin (20 units in 19 mL normal saline) (n=52)	375.7±292.3	254.1±185.4	84.4±20	79.8±31.5
Frederick, 2013	RCT	Randomized numbers table		Double	Jamaica	Single, university	February 2005–April 2005	Abdominal	50	Tourniquet intraoperatively, perivascular vasopressin (20 units in 19 mL normal saline) (n=25)	400 micrograms rectal misoprostol 30 min preoperatively, tourniquet intraoperatively, perivascular vasopressin (20 units in 19 mL normal saline) (n=25)	623±354	334±261		
Hashmi, 2021	RCT			Single	Pakistan	Single, university	January 2019–December 2019	Abdominal	100	Rectal placebo 1 h preoperatively (n=50)	400 micrograms rectal misoprostol 1 h preoperatively (n=50)	501.16±17.64	388.17±37.18		
Ihab Hassan, 2017	RCT	Computer generated random table	Sealed envelopes	None	Egypt	Single, university	August 2015– December 2016	Abdominal	60	2-0 vicryl for bilateral ascending uterine artery ligation intraoperatively (n=30)	400 micrograms vaginal misoprostol 1 h preoperatively (n=30)	438.43±124.79	447.4±133.32	81.33±21.09	80.83±24.25
Johnpaul, 2021	RCT			None	Nigeria	Multicenter	Not described	Abdominal	140	Tourniquet intraoperatively (n=66)	400 micrograms rectal misoprostol 1 h preoperatively, tourniquet intraoperatively (n=67)	583.5±186.2	522.6±127.9		
Kalogiannidis, 2011	RCT	Sequential	Sealed envelopes	Double	Greece	Single	February 2007– February 2009	Laparoscopic	67	Vaginal placebo 1 h preoperatively (n=34)	400 micrograms vaginal misoprostol 1 h preoperatively (n=30)	217±74	126±41	77±20	86±23
Khan, 2020	RCT	Computer-generated random table	Sealed envelopes	None	Pakistan	Single	July 2017– December 2018	Abdominal	50	Rectal placebo 30 min preoperatively (n=25)	800 micrograms rectal misoprostol 30 min preoperatively (n=25)	484.8±188	328.4±149	34.3	25.9
Maneerat, 2019	RCT	Computer generated random numbers	Sealed envelopes	Single	Thailand	Single, university	October 2016– August 2017	Abdominal, laparoscopic	46	Rectal placebo of 200 mg vitamin B_6_ 30 min preoperatively (n=24)	400 micrograms misoprostol PR 30 min preoperatively (n=22)	500±663	350±613	153.05	171.45
Mohamed, 2019	RCT	Random number generator, 1:1 allocation	Sealed envelopes	Double	Egypt	Single, university	October 2017– May 2018	Abdominal	75	Rectal placebo (n=25)	400 micrograms rectal misoprostol (n=25)	815.5±187.7	460.85±155.2	87±21.2	70.84±11.3
Mostafa-Gharabaghi, 2017	RCT	Randomized number table		Double	Iran	Single, university	January 10, 2014– February 28, 2015	Abdominal	70	Oxytocin infusion 30 units in 1,000 mL normal saline solution rate of 130 mL/h, started 20 min before incision (n=35)	400 micrograms vaginal misoprostol 1 h preoperatively (n=35)	589±49	401±48	93±2	82±3
Niroomand, 2015	RCT	Block randomization		Double	Iran	Single, university	September 2012– September 2013	Laparoscopic	90	Vaginal placebo of B_6_ 3 h preoperatively (n=40)	200 micrograms vaginal misoprostol 3 h preoperatively (n=40)	696±411	458±287	127±47	106±29
Nnagbo, 2023	RCT	Computer-generated random numbers	Sealed envelopes	None	Nigeria	Multicenter	February 2019– August 2019	Abdominal	126	Tourniquet intraoperatively (n=63)	400 micrograms vaginal misoprostol 1 h preoperatively, tourniquet intraoperatively (n=63)	583.5±186.2	522.6±127.91		
Ragab, 2014	RCT	Computer-generated random table	Sealed envelopes	None	Egypt	Single, university	January 2011–January 2013	Abdominal	69	400 micrograms vaginal misoprostol 1 h preoperatively (n=34)	800 micrograms vaginal misoprostol 3 and 1 h preoperatively (n=35)	200.2±18.8	101.4±25.5	35.4±5.6	25.8±4.1
Shafqat, 2019	RCT			None	Pakistan	Single, university	January 2018– December 2018	Abdominal	50	Tranexamic acid (10 mg/kg, maximum 1 g loading dose over 10 min followed by infusion of 1 mL/kg/min for duration of surgery) (n=25)	400 micrograms vaginal misoprostol 1 h preoperatively (n=25)	481±212	310±120		
Sharami, 2020	RCT	Random block sampling		Double	Iran	Single, university	January 2018–12/2018	Abdominal	94	Rectal placebo 1 h preoperatively (n=47)	400 micrograms rectal misoprostol 1 h preoperatively (n=47)	503.7±272.4	371.60±239.35	68.51±17.41	17.63±85.13
Son, 2019	RCT			Double	United States	Single		Robotic	79	Dilute vasopressin (n=42)	400 micrograms vaginal misoprostol, dilute vasopressin (n=32)	247	246	173.9	181.3
Srivastava, 2018	RCT	Computer-generated random table		Double	India	Single, university	March 2014– August 2015	Laparoscopic	60	Myometrial injection of dilute vasopressin (20 units in 100 mL normal saline) (n=30)	600 micrograms vaginal misoprostol 30 min preoperatively, myometrial injection of dilute vasopressin (20 units in 100 mL normal saline) (n=30)	206±101.2	139±96.7	135±60	120±2.5
Wetherell, 2022	RCT	Randomized numbers table	Sealed envelopes	Double	Australia	Single, university	September 2017– January 2021	Abdominal, laparoscopic	53	Myometrial injection of dilute vasopressin (20 units in 40 mL normal saline) (n=28)	400 micrograms sublingual misoprostol 30 min preoperatively, myometrial injection of dilute vasopressin (20 units in 40 mL normal saline) (n=25)	325±352	306±281	126±51	112±16

RCT, randomized controlled trial.

The sample sizes ranged from 50 to 80 participants (after accounting for withdrawals). The overall pooled average age was 34.9 years. The study by El Sharkwy et al^27^ enrolled an average older population (41 years), but this was within the SD of other studies. Similarly, El Sharkwy et al^27^ had a higher average body mass index (BMI) compared with other studies (31.5, which is categorized as obese). Although this was within the SD of other studies, it was clinically different. The pooled average BMI was 26.5 (which is in the overweight category).

The study by Afolabi et al^22^ had on average more reported leiomyomas per participant case than other studies that reported this variable (11 compared with less than five), but the overall uterus size was within the range of the other studies.^22,26,27,32,34,36,38–40,43,44^ For the studies that reported on mean maximum diameter of the largest leiomyoma (n=11), the Celik et al^26^ study had essentially double the average size of the leiomyomas of other studies (15 cm vs 8–9 cm).^23,24,26,27,32,34,36,37,41,43,44^ Their mean blood loss was not different from the majority of the studies.

The majority of studies (n=18, 75%) conducted open abdominal myomectomies only.^20-31,33,35,36,38–40^ Four studies used a laparoscopic approach, one of which used a robotic-assisted approach.^32,37,42,43^ Three studies used either open or laparoscopic approaches.^34,44^ Two studies did not describe the route of myomectomy^19,41^ (Table 1).

The risk of bias was variable. Sequence generation was typically random and computer generated, thus conferring a low risk of bias. Allocation concealment was typically done with sealed envelopes. Blinding was often a source of bias because of the unblinded nature of the interventions. Blinding of outcome assessors tended not to be described by studies. The studies consistently reported the data and outcomes that they said they would (Table 2).

**Table 2.** Risk of Bias by Study

First Author, Year	Sequence Generation	Allocation Concealment	Blinding of Participants or Personnel	Blinding of Outcome Assessors	Incomplete Outcome Data	Selective Outcome Reporting	Other Sources of Bias	Overall Risk of Bias
Abbas, 2019	Low risk	Low risk	Low risk	Unclear	Unclear	Unclear	Unclear	Unclear
Abbas, 2020	Unclear	Unclear	High risk	Unclear	Unclear	Low risk	Unclear	Unclear
Abdel-Hafeez, 2015	Low risk	Low risk	Low risk	High risk	Low risk	Unclear	Low risk	Unclear
Afolabi, 2019	Low risk	Low risk	High risk	Unclear	Low risk	Low risk	Unclear	Unclear
Alhalaby, 2021	Unclear	High risk	High risk	High risk	Unclear	Low risk	Unclear	High risk
Alhameedi, 2021	Unclear	Unclear	High risk	High risk	High risk	High risk	High risk	High risk
Ali, 2019	Unclear	Unclear	Low risk	High risk	High risk	Low risk	Unclear	High risk
Celik, 2003	Low risk	Low risk	Low risk	Unclear	Unclear	Unclear	Unclear	Unclear
El Sharkwy, 2016	Low risk	Low risk	High risk	High risk	Unclear	Low risk	Low risk	High risk
Frederick, 2013	Low risk	Low risk	Low risk	Low risk	Low risk	Low risk	Low risk	Low risk
Hashmi, 2021	High risk	Unclear	Unclear	Unclear	Unclear	Low risk	High risk	High risk
Ihab Hassan, 2017	Low risk	Low risk	High risk	Unclear	Low risk	Low risk	Low risk	Unclear
Johnpaul, 2021	Low risk	High risk	High risk	Unclear	Unclear	High risk	High risk	High risk
Kalogiannidis, 2011	Low risk	Low risk	Low risk	Low risk	Low risk	Low risk	Unclear	Low risk
Khan, 2020	Low risk	Low risk	Low risk	Low risk	Unclear	Low risk	Unclear	Unclear
Maneerat, 2019	Low risk	Low risk	Low risk	Low risk	Unclear	Low risk	Unclear	Unclear
Mohamed, 2019	Low risk	Low risk	Low risk	Low risk	Unclear	Low risk	Unclear	Unclear
Mostafa-Gharabaghi, 2017	Low risk	Low risk	High risk	Low risk	Low risk	Low risk	Unclear	Unclear
Niroomand, 2015	Low risk	Low risk	Low risk	Low risk	Low risk	Low risk	Unclear	Low risk
Nnagbo 2023	Low risk	Low risk	High risk	High risk	High risk	Unclear	High risk	High risk
Ragab, 2014	Low risk	Low risk	Low risk	Unclear	Unclear	Unclear	High risk	Unclear
Shafqat, 2019	High risk	High risk	High risk	Unclear	High risk	High risk	Unclear	High risk
Sharami, 2020	Low risk	Low risk	Low risk	Low risk	Unclear	Low risk	Unclear	Unclear
Son, 2019	Unclear	Low risk	Low risk	Unclear	Low risk	Low risk	Unclear	Unclear
Srivastava, 2018	Low risk	Low risk	Low risk	Low risk	Low risk	Low risk	Low risk	Low risk
Wetherell, 2022	Low risk	Low risk	Unclear	Low risk	Low risk	Low risk	Unclear	Unclear

**Fig. 2.**
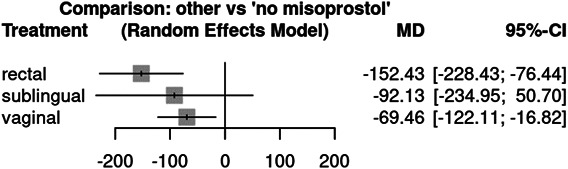
Meta-analysis mean reduction in blood loss by route of misoprostol administration compared to placebo under random effects model. MD, mean difference.

**Fig. 3.**
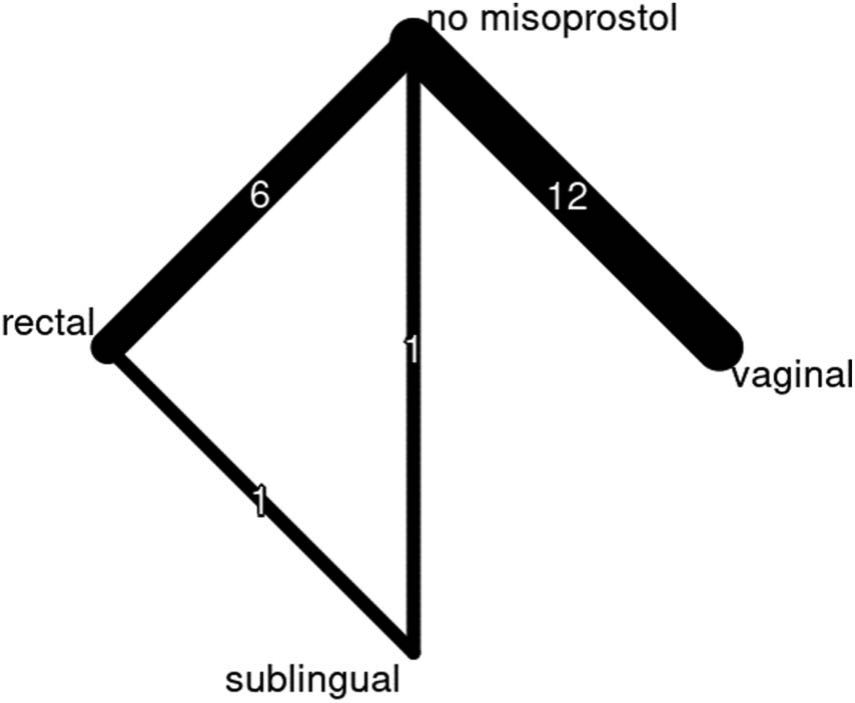
Network meta-analysis network diagram with thickness of lines representing number of studies included in the comparison.

The interventions ranged from variable doses of misoprostol, different routes of misoprostol, or misoprostol compared with a placebo, technique, or other medication (eg, vasopressin or tranexamic acid). All routes of misoprostol administration (rectal, sublingual, or vaginal) reduced blood loss compared with no misoprostol. Under the random-effects model (Fig. 2), rectal administration was associated with the largest mean reduction and was statistically significant (mean difference −152.43 mL, 95% CI, −228.43 to −76.44, *P*<.0001), followed by vaginal administration (mean difference −69.46 mL, 95% CI, −122.11 to −16.82, *P*=.0097). Sublingual administration did not have a statistically significant reduction under this model but still showed a mean reduction (mean difference −92.13 mL, 95% CI, −234.95 to 50.70, *P*=.206). Under the common-effects model, all routes of administration had statistically significantly reduced mean blood loss. More studies reported rectal administration compared with vaginal and sublingual administration (Fig. 3).

*Kaonga. Route of Misoprostol: Meta-analysis. O&G Open 2026*.

*Kaonga. Route of Misoprostol: Meta-analysis. O&G Open 2026*.

Rectal misoprostol significantly reduced the risk of having a blood transfusion postoperatively, whereas vaginal and sublingual misoprostol did not have a statistically significant reduced risk of a blood transfusion compared with placebo. Rectal misoprostol was associated with the least change in hemoglobin and hematocrit followed by vaginal administration compared with no misoprostol (Table 3). The sublingual route did not show a statistically significant drop.

**Table 3.** Network Meta-analysis Summary Table

	Effect (Common)	Effect (Random)	95% CI (Common)	95% CI (Random)	*P* (Common)	*P* (Random)	τ^2^	*I*^2^ (%)	*Q* Total	Q *P*	95% Prediction Interval
Blood loss (mL)											
Rectal	−116.43	−152.43	−124.25 to −108.60	−228.43 to −76.44	.000	.000	7,761.554	95.2	357.61	.000	−341.09 to 36.22
Sublingual	−99.07	−92.13	−137.55 to −60.58	−234.95 to 50.70	.000	.206	7,761.554	95.2	357.61	.000	−316.22 to 131.96
Vaginal	−100.57	−69.46	−109.81 to −91.33	−122.11 to −16.82	.000	.010	7,761.554	95.2	357.61	.000	−249.99 to 111.06
Operating time (min)											
Rectal	−15.21	−18.94	−18.71 to −11.71	−29.48 to −8.40	.000	.000	114.239	96.8	378.82	.000	−42.39 to 4.51
Sublingual	−14.00	−14.00	−28.39 to 0.39	−39.41 to 11.41	.057	.280	114.239	96.8	378.82	.000	−46.94 to 18.94
Vaginal	−8.89	−3.63	−9.62 to −8.16	−10.93 to 3.68	.000	.330	114.239	96.8	378.82	.000	−25.82 to 18.56
Transfusion events											
Rectal	−0.78	−0.79	−1.27 to −0.29	−1.33 to −0.25	.002	.004	0.080	11.1	13.50	.334	−1.56 to −0.01
Sublingual	0.69	0.69	−1.68 to 3.06	−1.74 to 3.13	.567	.577	0.080	11.1	13.50	.334	−1.80 to 3.19
Vaginal	−0.12	−0.11	−0.85 to 0.60	−0.88 to 0.65	.740	.772	0.080	11.1	13.50	.334	−1.06 to 0.83
Change in Hgb (g/dL)											
Rectal	−0.68	−0.74	−0.88 to −0.47	−1.17 to −0.30	.000	.001	0.268	79.6	63.65	.000	−1.84 to 0.37
Sublingual	−0.10	−0.10	−0.89 to 0.69	−1.39 to 1.19	.804	.879	0.268	79.6	63.65	.000	−1.74 to 1.54
Vaginal	−0.86	−0.60	−1.03 to −0.70	−1.02 to −0.17	.000	.007	0.268	79.6	63.65	.000	−1.70 to 0.51
Change in Hct (%)											
Rectal	−1.58	−1.18	−2.41 to −0.76	−3.94 to 1.58	.000	.402	5.282	90.1	60.30	.000	−6.47 to 4.10
Vaginal	−1.67	−1.38	−2.30 to −1.04	−3.53 to 0.76	.000	.206	5.282	90.1	60.30	.000	−6.37 to 3.61

Hgb, hemoglobin; Hct, hematocrit.

*P* is the pooled *P* value.

Similar to blood loss, rectal misoprostol significantly shortened operative time compared with no misoprostol (mean difference −18.94 minutes, 95% CI, −29.48 to −8.40, *P*<.001). Vaginal administration and sublingual administration also showed shorter operative times but were not significant in the random-effects model. Heterogeneity was high (τ^2^=114.24, *I*^2^=96.8%) (Table 3).

When we compared the same dose of misoprostol but different routes of administration, rectal misoprostol outperformed the vaginal and sublingual routes in reducing blood loss (Table 3).

## DISCUSSION

We evaluated the effectiveness of different routes of misoprostol administration in reducing blood loss during abdominal myomectomy. We found that all routes (rectal, vaginal, and sublingual) were associated with reductions in blood loss compared with no misoprostol, although this was not statistically significant across all routes. However, rectal administration appeared to be the most effective route, significantly reducing both intraoperative blood loss and risk of perioperative blood transfusion. Vaginal administration performed slightly better than sublingual administration, and although not statistically significant, both did seem to be superior to placebo. Of note, the sublingual administration analyses were limited by the number of studies assessing that route.

Previous systematic reviews have demonstrated that misoprostol decreases intraoperative blood loss at myomectomy. The Cochrane review in 2014 concluded that vaginal misoprostol reduced blood loss, although this was based on only two small randomized controlled trials (RCTs). Iavazzo et al^11^ also found benefit with vaginal misoprostol, but analyses were limited by the small number of studies included (five RCTs, of which three were included for blood loss analysis in their study). Wali et al^15^ restricted their analysis to open myomectomies and excluded studies that incorporated other blood loss reduction strategies (ie, vasopressin). Their findings confirmed a reduction in blood loss with misoprostol compared with placebo. More recently, Abu-Zaid et al^5^ included 16 RCTs (reflecting 945 participants) and reconfirmed findings from prior studies that misoprostol significantly reduced intraoperative blood loss (n=15, mean difference 180.2 mL, 95% CI, −224.04 to −136.35, *P* 0.001). However, none of these prior review studies directly compared routes of administration.

Recent practice guidelines from the AAGL reflect data from recent studies and encourage the use of misoprostol as a blood loss reduction strategy even in laparoscopic myomectomies.^45^

Our analysis expands on these prior studies by comparing routes directly with each other and including adjuvant medications and strategies. We also included both open and minimally invasive myomectomy approaches. This broader inclusion reflects contemporary practice trends and enhances generalizability while still confirming the overall benefit of misoprostol.

Pharmacokinetic studies provide useful context for our findings. The route of administration (sublingual, vaginal, rectal) influences onset, peak concentration, and duration of action. Oral, including sublingual, misoprostol has the highest peak plasma concentration and fastest onset but shortest duration, whereas vaginal and rectal routes provide more sustained plasma levels and can persist for at least 6 hours at higher concentrations.^3^ This pharmacologic profile likely explains why rectal and vaginal administration indicated a possible advantage in reducing blood loss compared with placebo, particularly for longer procedures such as myomectomies. For surgical planning, the typical 45- to 60-minute delay to peak concentration for the nonsublingual routes aligns well with preoperative preparation, supporting the administration of misoprostol in advance of abdominal entry.

Misoprostol has also been evaluated against other pharmacologic and surgical strategies. Some studies suggest that vasopressin in combination with misoprostol provides maximal benefit, although others show that misoprostol is comparable to mechanical methods such as pericervical tourniquet or uterine artery ligation.^4,25,36,40,46^ It is important to note that misoprostol is inexpensive and widely available and has a favorable side-effect profile compared with alternatives such as vasopressin or gonadotropin releasing hormone analogs. We were unable to assess misoprostol with and against other medications and techniques because there were too few studies to robustly incorporate adjuvants into the network meta-analysis; this presents an opportunity for future research.

Our review found that rectal misoprostol generally outranked vaginal and sublingual routes across several key outcomes compared with no misoprostol, although we could not ascertain the “best dose” because of limited number of studies evaluating different doses in our network meta-analysis. Evidence from two RCTs suggests higher doses (eg, 400 micrograms given twice preoperatively) may confer additional benefit, although these regimens were not consistently evaluated across the included trials.^19,39^ Vaginal misoprostol at 200 micrograms also appeared effective in one study, but the evidence base remains limited. Optimal timing remains approximately 1 hour before surgery to coincide with peak plasma concentration and sustained effect.

The strengths of this review include an inclusive search strategy, incorporation of both open and laparoscopic myomectomy approaches, and direct comparison of administration routes through network meta-analysis. However, limitations included heterogeneity in study populations, dosing regimens, surgical approaches, outcome reporting, and study quality. Inclusion of both laparoscopic and open procedures may increase variability, although our findings are consistent across surgical approaches. In addition, relatively few trials directly compared routes or doses, highlighting the need for further high-quality studies. In particular, very few studies investigated the sublingual route, limiting our interpretation of those data.

Focusing on the high heterogeneity across most of the outcomes, we also calculated a prediction interval; those results suggest that the effects could be negligible or nonexistent in a similar future study. We posit that this may be attributable to the variability across different populations and study conditions. Although the heterogeneity is high, the overall direction across the outcomes consistently shows benefits with rectal and, in some cases, vaginal misoprostol in reducing blood loss, decreasing operative time, and lowering transfusion events.

Misoprostol is effective in reducing blood loss during myomectomy, with rectal administration generally outperforming the other routes of administration, although these results should be interpreted with caution because of both the limited power to detect a difference and the limited evidence base of the sublingual route. Overall, our findings support the use of misoprostol as an effective and practical strategy to reduce blood loss during myomectomy. Given its low cost and safety profile, misoprostol represents an attractive option in both high- and low-resource settings. Future studies should focus on direct comparisons of routes, standardized dosing protocols, and evaluation in laparoscopic and robotic myomectomies, in which blood loss is typically lower.
